# Antizymotic Treatment of Diphtheria

**Published:** 1877-03

**Authors:** 


The Antizymotic Treatment of Diphtheria.—Dr. Pavesi describes, in the *Annali di Chimica Applic. alla Medicina*,, 1876 (abstract in *Annali Universali di Medicina*, August), a formula which he recommends in the treatment of diphtheria. If is founded on the antizymotic properties of chloral, salicylic acid, and the sulphites. It is as follows: ℞ Chloral hydrate, salicylic acid, glycerine, sulphite of soda, each 1½ parts; distilled water, 3½ parts; spirits of wine, 1 part. The whole is put into a strong glass vessel, which is closed, and exposed to a heat of 100° to 120° Fahr. for a few minutes, until the sulphite, silicylic acid, and chloral are completely evolved. A homogeneous solution is produced, which is filtered through bibulous paper, and preserved in a well-closed vessel. It is an oily, limpid, colorless liquid, having the odor of its constituent parts. It is insoluble in water. On the application of proper tests, the chloral, salicylic acid, sulphite of soda, and glycerine are found to be unchanged. Used both internally and externally, it is an energetic antiseptic, anti-fermentative, disinfectant, hæmostatic, and preservative, as well as a destroyer of parasitic organisms. Dr. Pavesi says that it may be used as an antiseptic, and also as a sedative, in a large number of diseases.—*London Med. Record.*

